# Expertise at Risk: Recent Policy Changes at HHS Advisory Committees

**DOI:** 10.1093/geroni/igaf122.3763

**Published:** 2025-12-31

**Authors:** Apoorva Rangan

**Affiliations:** Stanford University, Los Altos Hills, California, United States

## Abstract

Within the federal government, scientific advisory committees play a critical role in shaping evidence-based research priorities, clinical practice guidelines, and healthcare delivery for older people. There are over 1,000 advisory committees currently advising the federal government, 268 within the Department of Health and Human Services. Committee members are subject-matter experts who participate in committee work on a voluntary basis. As of July 2025, 79 committees have been terminated, and 23 are administratively inactive, representing a significant shift in governance and domain expertise across the Department.1 There have also been significant changes in the membership of several committees.2 This session will use the Advisory Committee on Immunization Practices (ACIP) as a paradigmatic case to describe the direct and indirect roles of scientific committees in guiding healthcare delivery and clinical practice.3, 4 We will also examine recent threats to the ACIP’s autonomy and outline potential consequences for clinicians, professional societies, and older patients. Data sources will include administrative notices of meeting postponements, CMS quality measures which incorporate ACIP recommendations, and legislation introduced by current members of Congress.5 We identify other committees related to aging which have faced interruptions, including the National Advisory Committee on Aging and identify the GSA’s role in restoring committee meetings. We advocate for HHS leadership and policy makers to preserve the ability of committees to function independently, promote certainty in guideline development, and improve stakeholder confidence. Finally, we address how organizations can advocate for these critical independent scientific committees and describe other routes to communicate science to policymakers.

